# Prediction of Welding Deformation and Residual Stress of a Thin Plate by Improved Support Vector Regression

**DOI:** 10.1155/2021/8892128

**Published:** 2021-03-03

**Authors:** Lei Li, Di Liu, Shuai Ren, Hong-gen Zhou, Jiasheng Zhou

**Affiliations:** School of Mechanical Engineering, Jiangsu University of Science and Technology, Zhenjiang 212003, China

## Abstract

Thin plates are widely utilized in aircraft, shipbuilding, and automotive industries to meet the requirements of lightweight components. Especially in modern shipbuilding, the thin plate structures not only meet the economic requirements of shipbuilding but also meet the strength and rigidity requirements of the ship. However, a thin plate is less stable and prone to destabilizing deformation in the welding process, which seriously affects the accuracy and performance of the thin plate welding structure. Therefore, it is beneficial to predict welding deformation and residual stress before welding. In this paper, a thin plate welding deformation and residual stress prediction model based on particle swarm optimization (PSO) and grid search(GS) improved support vector regression (PSO-GS-SVR) is established. The welding speed, welding current, welding voltage, and plate thickness are taken as input parameters of the improved support vector regression model, while longitudinal and transverse deformation and residual stress are taken as corresponding outputs. To improve the prediction accuracy of the support vector regression model, particle swarm optimization and grid search are used to optimize the parameters. The results show that the improved support regression model can accurately evaluate the deformation and residual stress of butt welding and has important engineering guiding significance.

## 1. Introduction

Welding is a process of forming permanent connection by heating or pressing the material of the workpiece to achieve the combination of atoms. Compared with riveted parts, castings, and forgings, welding products have the advantages of being lightweight, having a simple process, having low production cost, and having strong adaptability [1]. The welding technology is an indispensable processing method, which is widely used in aerospace, atomic energy, electronic technology, shipbuilding, construction, marine engineering, transportation and machinery manufacturing, and other industrial sectors [2–4]. Various welding deformations can occur in ship structures due to uneven heat and cold, local uncoordinated plastic strains, and welding residual stresses. Welding deformation and stress are important factors that affect the quality of hull construction and have a great impact on the welding performance, structural strength, toughness, aesthetics, and accuracy control of ship construction. This phenomenon is more obvious in thin plate welding [5]. Therefore, the research on prediction and control of welding deformation and residual stress of a thin plate has a very important engineering application value.

With the development of numerical analysis theory, the finite element method (FEM) has been adopted by many studies to predict welding deformation and residual stress. Since Ueda and Yamakawa analyzed the welding thermal stress and residual stress of butt and fillet welds by the thermo-elastic-plastic FEM, many researchers continued to develop this method for calculating the temperature, residual stress, and deformation [6]. At present, the FEM is also used in different welded joints and simple structure welds [7–9]. Liang et al. studied the welding deformation of aluminum alloy sheets by experiment and the TEP finite element method. The out-of-plane deformation predicted by the finite element method is in good agreement with the measured results [10]. Deng et al. used the finite element method to predict the welding deformation and residual stress of a thin plate and further investigated the influence of external constraints on welding deformation [11–14]. Shen et al. derived the stress amplification factor considering the influence of the preliminary deformation of the thin plate on the basis of the modified thick plate calculation formula. Then, with the traditional specification of the calculation, results were compared to verify the feasibility and effectiveness of the formula [15, 16]. Hu et al. established a finite element model to study the lateral bow and direction residual stress of s690 cold-formed thin-walled steel [17].

In many cases, the deformation and residual stress are simulated by FEM and then verified by experimental measurement. However, the FEM takes a long time and requires high computer performance and the implementation of experimental measurement needs high condition and cost [5]. Therefore, it is meaningful to setup a model which can predict the deformation and residual stress before welding. In the past decade, various forecasting models have emerged. He and Li applied a support vector machine (SVM) to quantitatively evaluate the welding quality [18]. Tian and Luo established an intelligent prediction model for welding deformation of T-joint fillet weld based on combinatorial optimization algorithms [19]. Katherasan et al. adopted the artificial neural network (ANN) algorithm to determine the approximate optimal value of welding process parameters and then used particle swarm optimization (PSO) to optimize the process parameters [20]. Edwin and Kumanan discussed the application of the evolutionary fuzzy support vector regression model in welding residual stress prediction [21]. Ansaripour et al. uses the ANN algorithm and multiobjective optimization algorithm to minimize deformation and residual stress during submerged arc welding [22]. Rong et al. analyzed the influence of the weld profile on angle distortion and predicted the angle distortion of gapless tungsten inert gas arc weld using the neural network [23]. Mathewa et al. established the prediction model of residual stress distribution of austenitic stainless steel girth-welded pipe by using the artificial neural network [24]. Sagai et al. successfully used process parameters to predict mechanical properties by using the stochastic and nonlinear parallel machining neural network model [25]. Das et al. used the machine learning algorithm to predict the welding residual stress of stainless steel [26]. Mathew et al. used the fuzzy neural network (FNN) to predict the welding residual stress of pressure vessels and achieved good results [27]. Koo et al. proposed a prediction model of residual stress in the welding area based on improved support vector regression [28].

In this paper, the corresponding inherent deformation and residual stress are obtained under different values of voltage, current, speed, and plate thickness. Based on the experimental data, a prediction model of welding deformation and residual stress of a thin plate based on particle swarm optimization and grid search-improved support vector regression (PSO-GS-SVR) was established. Then, the predicted results of the PSO-GS-SVR model are compared with the experimental results. The results show that there is a high degree of consistency between the predicted results and the experimental measurements. The research shows that the improved support regression model can more accurately evaluate the welding deformation and residual stress of butt welding than the original model.

The rest of this paper is arranged as follows.  Section 2 shows that the deformation and residual stress corresponding to different welding parameters are obtained by experiments.  Section 3 introduces the related algorithm theory. In Section 4, an improved prediction method of support vector regression is established. The optimized SVR model was evaluated by comparing the prediction results with the experimental results, which is shown in Section 5. Finally, in Section 6, this paper is summarized and the future research direction is pointed out.

## 2. The Experimental Procedure

In this experiment, two similar V-joint planes of 300 × 150 mm plates were selected. The workpiece material is AH32, which is high-strength steel. The chemical composition and mechanical properties are shown in Table 1. Due to the small welding deformation and residual stress, CO_2_ gas-shielded welding is adopted, which is especially suitable for thin plate welding. The process parameters, namely, voltage (*U*), current (*A*), speed (*S*), and plate thickness (*T*), are varied in the range of 25–31 V, 180–210 A, 5–8 mm/s, and 3-6 mm, respectively. The detailed combination of the test process parameter design is shown in Table 2. Then, the transverse deformation and longitudinal deformation of welding are measured by a coordinate measuring machine (CMM). The residual stress of the workpiece was measured by X-ray diffraction.

### 2.1. Measurement of Deformation

**Figure 1:**
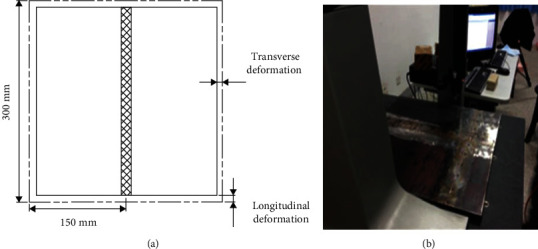
Welding deformation measurement. (a) Longitudinal and transverse deformation. (b) Experimental measurements.

In order to accurately measure the welding deformation value, the deformation results of V-shaped groove components after welding were measured by a coordinate measuring machine. The welding component is placed on the measuring platform, and the measuring platform is used as the zero-value point of bending deformation. Then, the transverse deformation and longitudinal deformation are obtained by measuring the marked measuring points on the workpiece surface step by step. Transverse deformation is the deformation perpendicular to the direction of the weld; longitudinal deformation is the deformation parallel to the direction of the weld, as shown in Figure 1(a). The deformation measurement process is shown in Figure 1(b).

### 2.2. Measurement of Residual Stress

**Figure 2:**
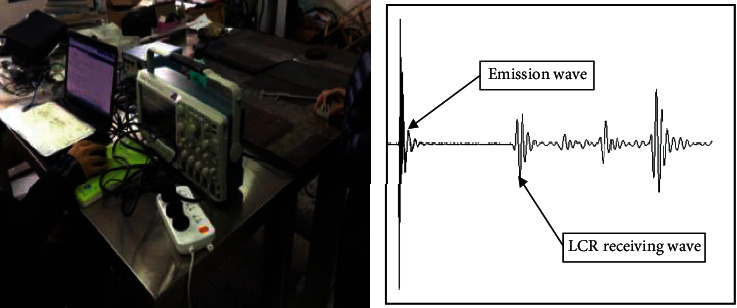
Measurement process of residual stress.

At present, there are many methods to measure welding residual stress. According to the different forms of residual stress measurement, it can be divided into destructive testing and nondestructive testing (NDT) methods [29, 30]. Nondestructive testing has been widely used for its advantages of not damaging components, such as magnetic measurement, ultrasonic measurement, and X-ray diffraction measurement [31–38]. In this paper, the ultrasonic method is selected for measurement. Because the residual stress perpendicular to the weld direction has a great influence on the structural strength, the point perpendicular to the weld direction on the half-width line of the workpiece is taken as the residual stress measurement point [39, 40].  Figure 2 shows the measurement process of residual stress.

### 2.3. Experimental Dataset

Through the above experimental measurement, this study consists of 24 groups of different experimentally measured datasets. The dataset is divided into two parts. The left half is for the different *U*, *I*, *S*, and *T* process parameters. The right half is the deformation values and residual stresses corresponding to the different process parameters. All the experimental results are shown in Table 3.

## 3. The Related Algorithm Theory

### 3.1. Support Vector Regression

To solve the problem of pattern recognition, the support vector machine (SVM) is proposed by Vapnik [41]. Later, the insensitive loss function is introduced and applied to the regression estimation of nonlinear systems, which forms support vector regression (SVR). The basic theory of SVR is to map the dataset to a high-dimensional space in the way of nonlinear transformation and then regress in this space to fit a continuous function to minimize the loss function. The goal is to find an optimal classification surface that minimizes the error of all training data, in order to achieve better fitting performance and generalization ability for unknown samples. The SVR has many unique advantages in solving a small sample, nonlinear, high-dimensional pattern recognition, and so on. It has been gradually applied to the prediction of welding deformation and residual stress. The learning ability and generalization ability of SVR are determined by the regularization coefficient *C* and the related parameters of kernel function. The basic model is as follows [42]:

(1) $$\begin{array}{c} f\left(x\right)=\omega \cdot \varphi \left(x\right)+b, \end{array}$$

where *φ*(*x*) represents the nonlinear mapping of the input sample space to the feature space, *ω* is the weight coefficient, and *b* is the offset term. The loss function is obtained by minimizing *ω* and *b*.

(2) $$\begin{array}{c} R\left(x\right)=\frac{1}{2}{\left\|\omega \right\|}^{2}+C\left(\overset{l}{\underset{i=1}{\sum }}\mu_{i}\left(\xi_{i}+{\xi_{i}}^{\prime }\right)\right), \end{array}$$

bound by formula (3) as follows:

(3) $$\begin{array}{c} \left\{\begin{array}{c} f\left(x_{i}\right)-y_{i}\leq \varepsilon +{\xi_{i}}^{\prime },\\ y_{i}-f\left(x_{i}\right)\leq \varepsilon +\xi_{i},\\ \xi_{i},{\xi_{i}}^{\prime }\geq 0,\\ \;i=1,2,\cdots ,l, \end{array}\right. \end{array}$$

where *l* represents the total of training data, *C* denotes the regularization coefficient, *ε* is the insensitive coefficient, *ξ*_*i*_ and *ξ*_*i*_′ are the relaxation variables, and (1/2)‖*ω*‖^2^ is the confidence risk of the model. The SVR model is as follows:

(4) $$\begin{array}{c} f\left(x\right)=\overset{l}{\underset{i=1}{\sum }}\left(a_{i}-{a_{i}}^{\prime }\right)K\left(x,x_{i}\right)+b, \end{array}$$

where the coefficient *a*_*i*_ − *a*_*i*_′ is not equal to zero and is solved using the quadratic planning technique, the corresponding training data vectors are called support vectors, and *K*(*x*, *x*_*i*_) is the kernel function.

For different kernel functions, the types and quantities of parameters that need to be determined are different. Since the RBF kernel function has the advantages of less parameters and nonlinear prediction, the RBF function is selected as the kernel function of the SVR regression model. The expression of the RBF kernel function is shown in formula (5) as follows:

(5) $$\begin{array}{c} K\left(x,x_{i}\right)=\exp\left(-\frac{\left\|x-\left.x_{i}\right\|\right.}{2g^{2}}\right), \end{array}$$

where *g* is the width parameter of the RBF kernel function, *x* is any input sample vector, and *x*_*i*_ is the center of the Gaussian RBF kernel function.

Therefore, it is important to find the optimal regularization parameter *C* and the width of kernel function *g*.

### 3.2. Particle Swarm Optimization

Particle swarm optimization (PSO), developed by Kennedy and Eberhart, is an evolutionary computing technology derived from the study of bird predation behavior [43]. Particle swarm optimization (PSO) has a faster convergence speed and less adjustable parameters, so it is easy to implement. Therefore, it is often used in combination with other algorithms to optimize parameters. The specific algorithm flow is as follows:

**Step 1 .**

Initialize the particle swarm optimization. Set the maximum iteration number, the maximum velocity of particles *v*_max_, the position of the space *x*_*i*_, the particle swarm optimization modulus *N*, and the initial flight speed *v*_*i*_.

**Step 2 .**

Define the moderation function. First, calculate the adaptation value for each particle and find the individual optimal solution from it. Then, the global optimal value of a group is found from the individual optimal solution. Finally, the global optimal value is compared with the historical global optimal value and updated.

**Step 3 .**

The velocity and position of particles are updated as shown in formula (6) as follows:

(6) $$\begin{array}{c} v_{i}=\omega *v_{i}+c_{1}*r_{1}*i\left(p_{id}-x_{i}\right)+c_{2}*r_{1}*\left(p_{gd}-x_{i}\right),\;i=1,2,\cdots ,N,\\ x_{i}=x+v_{i},\;i=1,2,\cdots ,N, \end{array}$$

where *c*_1_, *c*_2_ is the learning factor, *ω* is the inertia factor, *r*_1_, *r*_2_ ∈ (0, 1) is the random value, *p*_*id*_ is the particle local optimal position, and *p*_*gd*_ is the global optimal position.

**Step 4 .**

If the global optimal solution position satisfies the minimum limit or reaches the maximum iteration number, it will be the output to the particle with optimal fitness.

Because particle swarm optimization has the advantages of good global search ability, fast convergence speed, and less adjustable parameters, it can find the most suitable regularization parameters *C* and RBF parameter *g* of SVR more quickly and efficiently.

### 3.3. Grid Search

Grid search (GS) is widely used in support vector machines for parameter search optimization [44]. It can perform comprehensive search in parallel, and its computational complexity is very prominent. Since the RBF function is chosen as the kernel function of SVR, only the optimal values of *C* and *g* had to be determined. This method maps all combinations of these two parameters to a 3D grid, and then, solves all solutions and obtains the optimal combination. Finally, the groups of *C* and *g* with the highest prediction accuracy of cross-validation of the training set are selected as the optimal parameter combination.

## 4. The SVR Model Improved by the PSO and GS

**Figure 3:**
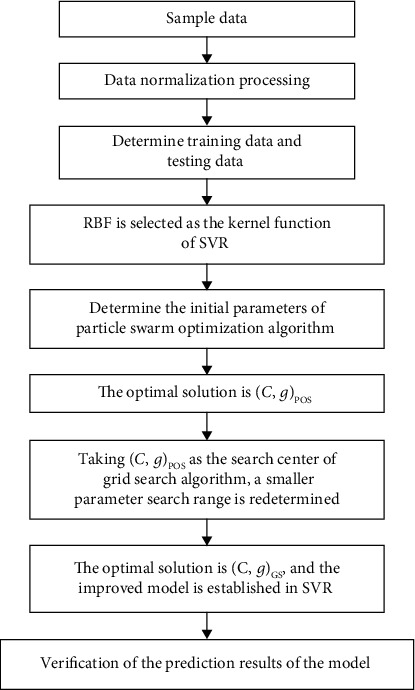
The main flow of the PSO-GS-SVR model.

In this paper, an improved SVR model is proposed by exploiting the advantages of particle swarm optimization and grid search. In the early stage of the algorithm, the particle swarm optimization (PSO) is used for fast rough search in a large range, and then, the population optimal solution (not necessarily the global optimal one) is found. Taking the optimal solution of the population as the initial position, the coordinate system grid of relevant parameters is established, and then, the grid search (GS) is used to search a small step between the initial positions of a small cell, which can avoid a large number of previous invalid operations. In this paper, an improved support vector regression (PSO-GS-SVR) deformation prediction model is developed to predict the weld deformation and residual stresses in butt joints with weld speed, weld current, weld voltage, and plate thickness as inputs. The improved support vector regression prediction model consists of three main steps, namely, data preprocessing, prediction model implementation, and resultant prediction validation. The main flow of the proposed PSO-GS-SVR model is shown in Figure 3.

### 4.1. Data Preprocessing

#### 4.1.1. Data Normalization Processing

In order to eliminate the difference between dimensions, the sample data is normalized and mapped to [0, 1]; the formula is as follows:

(7) $$\begin{array}{c} \bar{x}=\frac{x-x_{\min}}{x_{\max}-x_{\min}}. \end{array}$$

#### 4.1.2. Data Classification

The first 16 groups of data in Table 1 are used as training data, and the remaining 8 groups of data are used as test data.

### 4.2. Implementation of the Model

**Figure 4:**
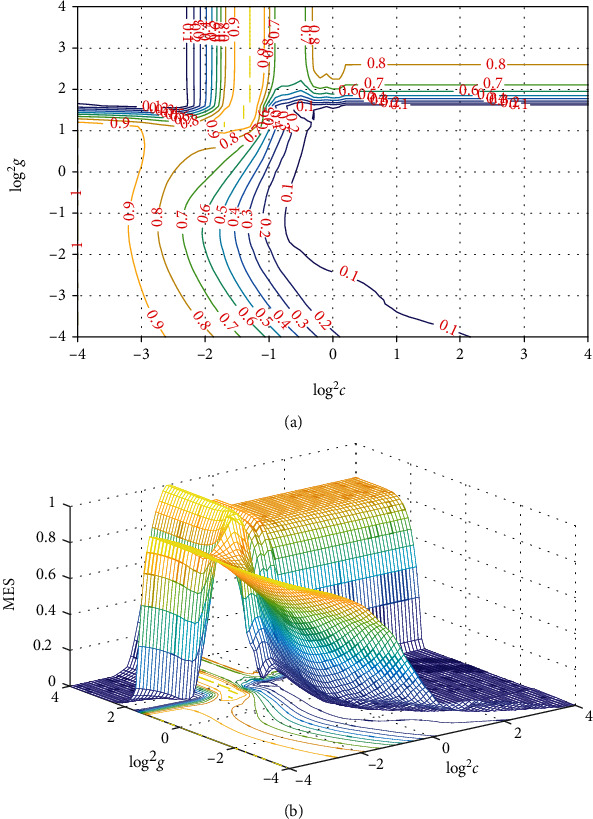
Result chart of refined parameter selection. (a) Contour map. (b) 3D view plot.

The main implementation steps are as follows:

1. Set initial boundary conditions. Particle swarm optimization (PSO) is used to optimize the width *g* and regularization coefficient *C* of the kernel function. The parameters involved include population size *m*, inertia weight *w*, acceleration constants *c*_1_ and *c*_2_, maximum velocity *V*_max_, and maximum algebra *G*_max_. It is determined that the population number is 20 and the population derivation algebra is 100. Because this is the first fast search, the search interval of parameter *C* can be set a little larger. Select the interval of two parameters as a learning factor. After all the preparatory work is completed, the first parameter optimization process can be started
2. After getting the optimal parameters of PSO, it is marked as (3.151, 0.624)_POS_. According to the optimal parameters, the grid of the coordinate system is established with the parameters as the central starting point and the second fine mesh search of the small step length is carried out. Since the step size of traditional grid search method is generally 0.1, the step size is set to 0.05 in the small step fine search. Set the inter cell as [2^−4^, 2^4^]. After a small step search, the optimal solution is obtained as shown in Figure 4, which is recorded as (3.732, 0.144)_ms_
3. The optimal parameters are substituted into the SVR model and its kernel function to establish the prediction model of improved support vector regression

## 5. Results and Discussion

In this section, the particle swarm optimization (PSO) and grid search(GS) optimized SVR model was evaluated by comparing the prediction results with the experimental results. Meanwhile, the accuracy of the optimized SVR model and the nonoptimized SVR model is also compared.

**Figure 5:**
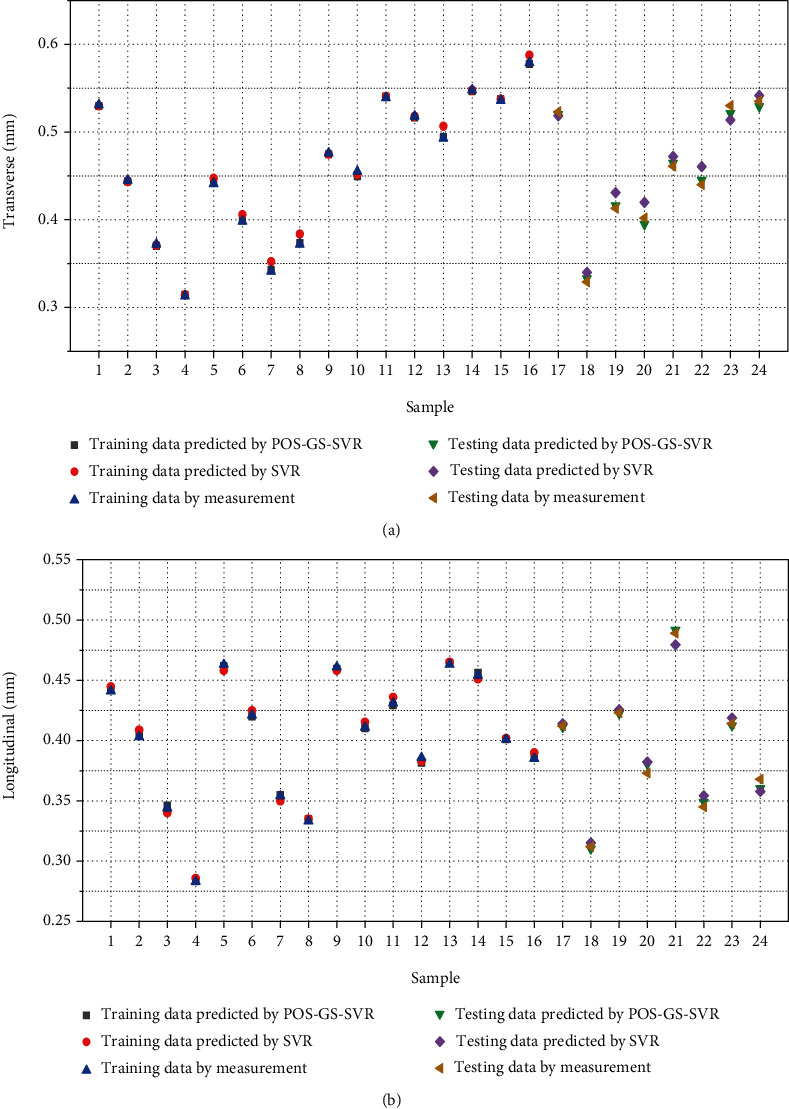
Comparison of predicted and experimental deformation values. (a) Transverse deformation. (b) Longitudinal deformation.

**Figure 6:**
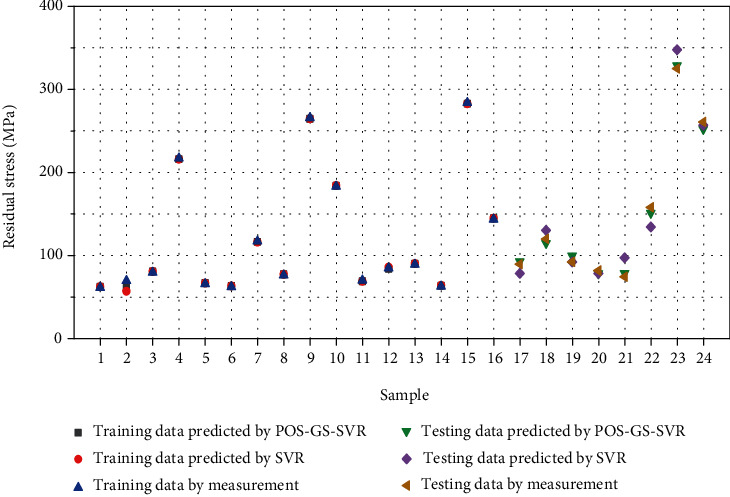
Comparison of predicted and experimental residual stress values.

The prediction results of welding deformation and residual stress are shown in Figures 5 and 6, respectively. Among them, the black “■” is the training data predicted by the PSO-GS-SVR model, the red “●” represents training sample data predicted by the SVR model, the blue “▲” stands for the training data of experimental measurement, the green “▼” means the testing data predicted by the PSO-GS-SVR mode, the violet “◆” denotes the testing data predicted by the SVR model, and the yellow “◀” indicates the testing data of experimental measurement. When the PSO-GS-SVR model is used, there is a small difference between the predicted values of weld deformation (as shown in Figure 5) and residual stress (as shown in Figure 6) and the experimental values. Conversely, the predictions of the SVR model are more inaccurate. In conclusion, the proposed PSO-GS-SVR model can improve the prediction accuracy.

In order to further evaluate the prediction performance of the model, we choose three indexes: coefficient of determination (*R*^2^), mean absolute percentage error (MAPE), and mean square error (MSE). These formulas are as follows:

(8) $$\begin{array}{c} R^{2}=\frac{\sum_{i=1}^{n}{\left({\overset{\wedge }{y}}_{i}-{\bar{y}}_{i}\right)}^{2}}{\sum_{i-1}^{n}{\left(y_{i}-{\bar{y}}_{i}\right)}^{2}},\\ \text{MAPE}=\frac{1}{n}\overset{n}{\underset{i=1}{\sum }}\left(\left|\frac{{\hat{y}}_{i}-y_{i}}{y_{i}}\right|\right)\times 100\%,\\ \text{MSE}=\frac{1}{n}\overset{n}{\underset{i=1}{\sum }}{\left(y_{i}-{\overset{\wedge }{y}}_{i}\right)}^{2}, \end{array}$$

where ${\hat{y}}_{i}$ and *y*_*i*_ denote the predicted values and the experimental values and ${\bar{y}}_{i}$ represents the mean value of experimental data. *n* is the number of samples.

The statistical results shown in Table 4 give the *R*^2^, MAPE, and MSE of deformation and residual stress. Combined with Figures 5 and 6, the two models are consistent with the overall trend of experimental data. However, further comparison between *R*^2^, MAPE, and MSE shows that the PSO-GS-SVR model is superior to the SVR model in all three indicators, no matter the testing data or training data. The coefficient of determination approaches is close to 1, indicating a very high degree of a match existing between the predicted values and experimental values. The value of MSE can evaluate the change degree of data. The MSE value tends to be 0, which means that the prediction model has good accuracy. The MAPE value is very small, indicating that the model is stable. Therefore, the proposed PSO-GS-SVR model is feasible to predict the deformation and residual stress of butt welding of thin plates.

## 6. Conclusion

This paper studies the development of particle swarm optimization and grid search-optimized SVR model for welding deformation and residual stress of butt joint sheets, and the conclusion is as follows:

1. The improved support vector regression model was developed for the prediction of welding deformation and residual stress of butt welding
2. The transverse deformation, longitudinal deformation, and residual stress corresponding to different values of welding voltage, welding current, welding speed, and plate thickness were measured by experiments
3. The experimentally obtained measurements are used to train the particle swarm and grid search-improved support vector machine model, which can predict the weld deformation and residual stress well. The results show that the PSO-GS-SVR model has excellent generalization capability
4. Through the comparison of the three indicators, the PSO-GS-SVR model has better prediction accuracy than the SVR. It provides an effective method to predict welding deformation and residual stress of a thin plate

The current research object is simple butt welding. Considering the diversity of welded joints, our next research work is deformation prediction of a complex welding structure.

## Figures and Tables

**Table 1:** The chemical composition and mechanical properties of AH32.

C	Mn	Si	S	P	Yield strength (MPa)	Tensile strength (MPa)	Elongation (%)
≤0.18	0.70~1.60	0.10~0.50	≤0.04	≤0.04	315	440~590	22

**Table 2:** Welding parameters.

Parameter	Units	Notation				
Voltage	V	*U*	25	27	29	31
Current	A	*I*	180	190	200	210
Speed	mm/s	*S*	5	6	7	8
Plate thickness	mm	*T*	3	4	5	6

**Table 3:** Experimental results.

Experiment number	Voltage (V)	Current (A)	Speed (mm/s)	Plate thickness (mm)	Transverse deformation(mm)	Longitudinal deformation (mm)	Residual stress (MPa)
1	25	180	5	3	0.531	0.441	61.35
2	25	190	6	4	0.445	0.403	69.78
3	25	200	7	5	0.372	0.344	79.67
4	25	210	8	6	0.313	0.283	217.08
5	27	180	6	5	0.441	0.463	65.78
6	27	190	5	6	0.398	0.421	62.23
7	27	200	8	3	0.341	0.354	117.36
8	27	210	7	4	0.372	0.333	76.27
9	29	180	7	6	0.476	0.461	265.80
10	29	190	8	5	0.455	0.411	183.03
11	29	200	5	4	0.539	0.431	70.23
12	29	210	6	3	0.518	0.386	84.98
13	31	180	8	4	0.493	0.463	89.23
14	31	190	7	3	0.548	0.454	62.82
15	31	200	6	6	0.536	0.401	283.97
16	31	210	5	5	0.579	0.385	143.63
17	25	190	5	3	0.523	0.412	89.67
18	25	210	7	3	0.329	0.312	119.90
19	27	180	8	4	0.413	0.423	92.27
20	27	200	7	4	0.402	0.373	81.89
21	29	180	5	5	0.461	0.489	74.56
22	29	210	7	5	0.440	0.345	158.02
23	31	190	8	6	0.530	0.414	325.09
24	31	210	6	6	0.535	0.368	260.82

**Table 4:** Prediction performance evaluation of the PSO-GS-SVR model.

		Transverse deformation			Longitudinal deformation			Residual stress		
		*R* ^2^	MAPE (%)	MSE	*R* ^2^	MAPE (%)	MSE	*R* ^2^	MAPE (%)	MSE
PSO-GS-SVR	Training	0.9931	0.5324	0.00001	0.9911	1.1496	0.00005	0.9967	1.9183	6.2346
	Testing	0.9677	1.2437	0.00004	0.9763	2.2527	0.00014	0.9745	4.3015	32.3187
SVR	Training	0.9281	1.5391	0.00010	0.9450	3.7545	0.00023	0.9809	2.2783	11.4254
	Testing	0.8619	4.2559	0.00044	0.9064	5.8612	0.00043	0.8939	9.9153	115.3360

## Data Availability

All the data used to support the findings of this study are included within the article.
